# Prenatal Exposure to Persistent Organic Pollutants Is Associated with Altered Lipid and Metabolomic Profiles in Mothers and Newborns

**DOI:** 10.1021/envhealth.6c00104

**Published:** 2026-06-09

**Authors:** Samira Salihovic, Tim Sinoja, Suvi M. Virtanen, Matej Orešič, Mikael Knip, Tuulia Hyötyläinen

**Affiliations:** † School of Medical Sciences, Faculty of Medicine and Health, 6233 Örebro University, Örebro 2026, Sweden; ‡ School of Science and Technology, Örebro University, Örebro SE-701 82, Sweden; § Department of Public Health, 3837 Finnish Institute for Health and Welfare, Helsinki FI-00271, Finland; ∥ Unit of Health Sciences, Faculty of Social Sciences, Tampere University, Tampere FI-33100, Finland; ⊥ Center for Child Health Research, Tampere University and Tampere University Hospital and Wellbeing Services County of Pirkanmaa, Tampere FI-33100, Finland; # Turku Bioscience Centre, University of Turku and Åbo Akademi University, Turku FI-20520, Finland; ¶ Department of Life Technologies, University of Turku, Turku FI-20014, Finland; ∇ Research Program for Clinical and Molecular Metabolism, Faculty of Medicine, University of Helsinki, Helsinki FI-00290, Finland; ○ Pediatric Research Center, Children’s Hospital, University of Helsinki and Helsinki University Hospital, Helsinki FI-00014, Finland

**Keywords:** persistent organic pollutants, lipidomics, metabolomics, pregnancy, cord blood

## Abstract

Maternal exposure to persistent organic pollutants (POPs) has been linked with adverse pregnancy and birth outcomes, yet the underlying biological pathways involved remain poorly characterized. In this study, we examined associations between POP concentrations in maternal plasma and cord blood and metabolomic and lipidomic profiles of pregnant women and their newborns and further assessed relationships with neonatal birth weight. A total of 257 mother–infant dyads were included. Concentrations of polychlorinated biphenyls (PCBs) and organochlorine pesticides (OCs) were quantified using gas chromatography coupled with atmospheric pressure tandem mass spectrometry (GC–MS/MS). Metabolomic profiling was performed using gas chromatography coupled with quadrupole time-of-flight mass spectrometry (GC–QTOFMS) and ultrahigh-performance liquid chromatography–QTOFMS (UHPLC–QTOFMS). In maternal plasma, POP exposure was predominantly associated with variations in free fatty acids, phospholipids, and triacylglycerols. The strongest associations were observed for *p*,*p*′-DDE and *trans*-nonachlordane, with *p*,*p*′-DDE also positively associated with the gut microbiota-derived metabolite indole-3-propionic acid. Several PCB congeners (74, 163, 170, and 180) showed pronounced associations with individual lipid species, particularly those containing polyunsaturated or monounsaturated fatty acyl chains. Although no significant associations were observed between POP exposure and neonatal birth weight, POP exposure was significantly associated with the cord blood metabolome, characterized by predominantly inverse associations with polyunsaturated fatty acid-containing lipids. Overall, these findings provide insights into metabolic pathways potentially linking maternal POP exposure with alterations in lipid and metabolomic profiles during pregnancy and early life.

## Graphical abstract

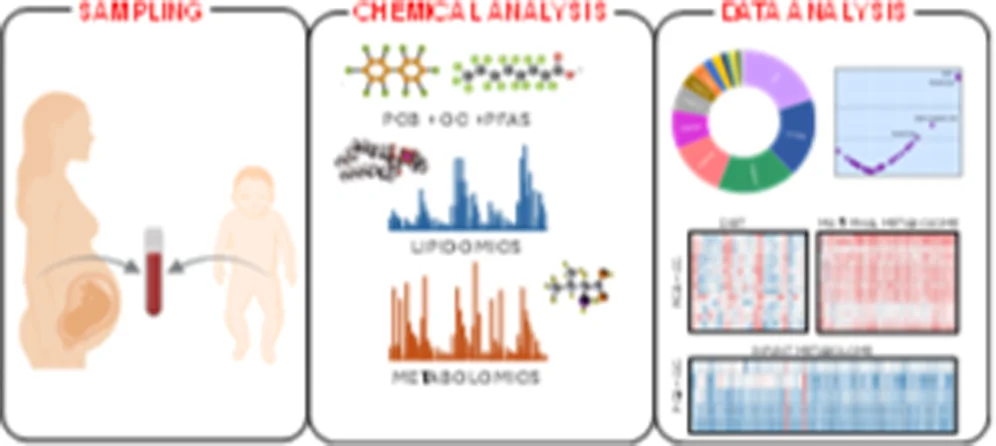

## 1 Introduction

Persistent organic pollutants (POPs) constitute a large group of halogenated chemicals that have entered the environment through production and application in various industrial and agricultural processes. POPs have been released to the environment for decades, thus, their detection in the environment (e.g., air, water, soil, biota) and humans (e.g., blood, adipose tissue, breast milk) is not a new issue. Nevertheless, they continue to pose a hazard for environmental and human health because of their well-recognized persistence, bioaccumulation, and toxic potential.^1,2^ The main exposure source of POPs in the general population is POP residues in food. During pregnancy, maternal POPs are transferred to the fetus via the placenta and later to the infant via breast milk.^3,4^

Growing body of evidence indicates that maternal exposure to endocrine-disrupting chemicals, e.g., polychlorinated biphenyls (PCBs) and organochlorine (OC) pesticides that can readily cross the placenta may not only influence maternal pregnancy outcomes but also neonatal outcomes. Epidemiological studies reported that increased maternal exposure to OC pesticides and PCBs is associated with altered maternal thyroid function,^5^ thus having potential implications for the development and health outcomes of the offspring. Organochlorine (OC) pesticides and PCBs have also been associated with alterations in placental DNA methylation, potentially contributing to prenatal POP toxicity and changes in neonatal anthropometric measures.^6^ Previous studies also suggest a link between increased maternal exposure to organochlorine chemicals and altered fetal growth,^7^ where associations with both low and high birth weights were reported.^8,9^ Moreover, high prenatal exposure to *p*,*p*’-DDE which is the main metabolite of DDT, has been linked with increased infant growth, while postnatal PCB-153 exposure has been associated with decreased infant growth, thus suggesting that different pollutants may have diverging or even opposing effects on neonatal anthropometric measures.^10^

The pathways through which POP exposure influences maternal, pregnancy, and neonatal outcomes remain incompletely characterized but may include molecular changes in maternal metabolism with implications for placental and fetal function. Thus, metabolomics approaches that enable the comprehensive assessment of a broad range of endogenous and exogenous metabolites in biofluids and tissues hold great potential for uncovering and identifying perturbed metabolic pathways.^11,12^ The main objectives of our study were to assess (a) the associations of exposures to environmental chemicals with metabolic profiles in mothers from mother-child cohort (*n* = 257) and cord blood of their offsprings by integrating targeted measurements of POP exposures from maternal plasma with comprehensive metabolomics and lipidomics profiling of both maternal plasma and cord blood and (b) the associations of prenatal POP exposures with neonatal birth weight.

## 2 Methods

The selection of the study participants and the analyses done are shown in Figure 1.

**Figure 1:**
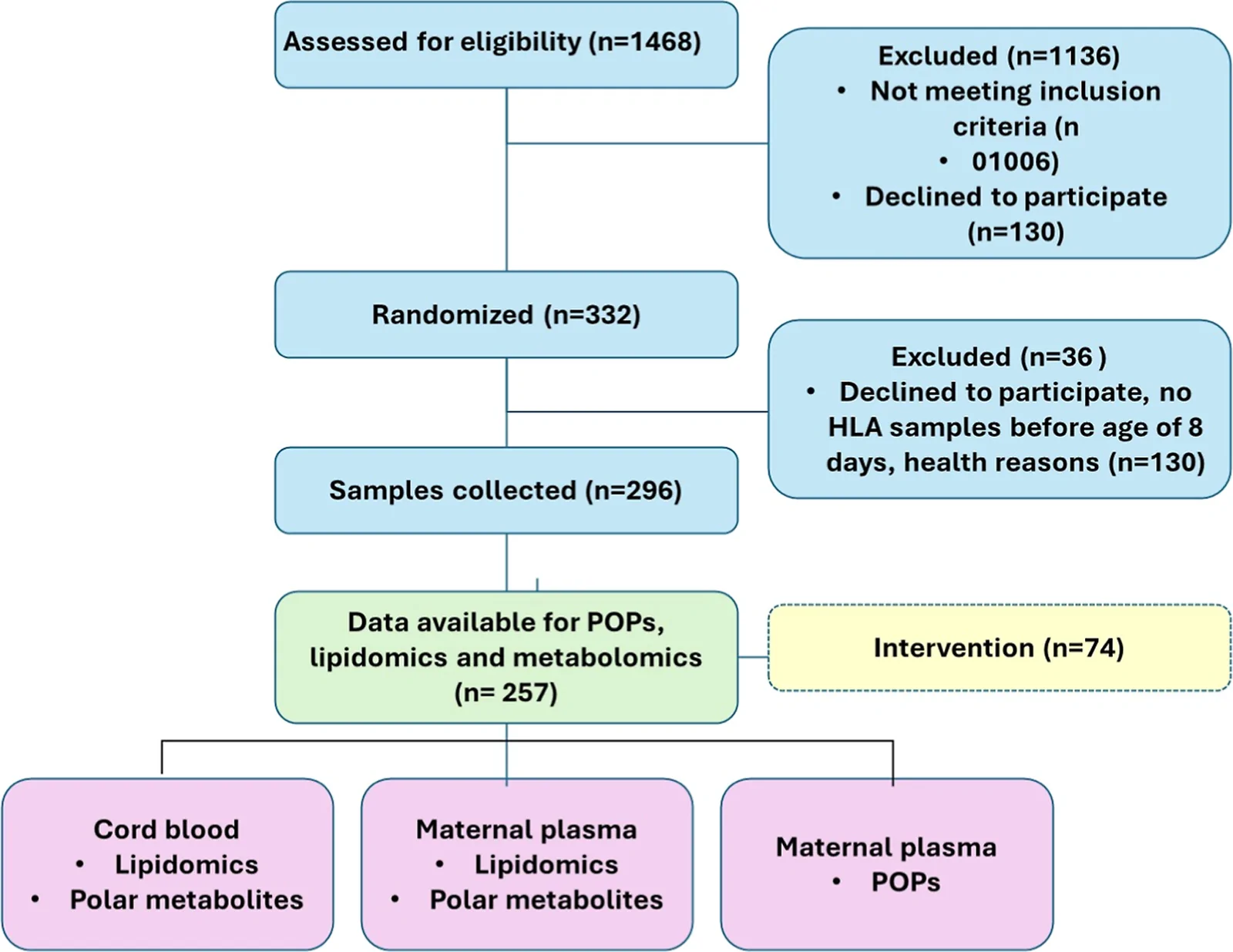
Selection of study participants and the analyses performed in the study.

### 2.1 Study Participants

Pregnant women were recruited from Finland between 2013 and 2015 as part of the EDIA (Early Dietary Intervention and Later Signs of Beta-Cell Autoimmunity: Potential Mechanisms) study (ClinicalTrials.gov: NCT01735123). Written informed consent was signed by the parents to permit analysis of their Human Leukocyte Antigen (HLA) genotype to exclude infants without possible HLA-conferred susceptibility to type 1 diabetes (T1D). At that point, 68% of the infants to be born were excluded from the follow-up. Separate informed consent was obtained from eligible parents at the beginning of the third trimester to analyze the offspring’s genotype and to continue in the intervention study. Blood samples were collected from 257 pregnant mothers at two time points (at the beginning of third trimester and at delivery) and pooled. Cord blood was also collected. Maternal diet during pregnancy was assessed by validated, semiquantitative food frequency questionnaire.^13^ Individual food and nutrient intakes were calculated using the national food composition database Fineli (https://fineli.fi/fineli/en/index). Missing data was imputed using median values.

### 2.2 Analysis of Persistent Organic Pollutants

Sample preparation and extraction of persistent organic pollutants (POPs) from maternal plasma were performed as previously described.^14,15^ Briefly, plasma samples were spiked with a mixture of 13C-labeled internal standards, including selected PCBs, OCDD, and PBDE-47. PCBs 81, 114, and 178 were used as recovery standards. Samples were extracted and purified using Oasis HLB solid-phase extraction followed by sulfuric acid-modified silica cleanup prior to instrumental analysis.

Instrumental analysis was performed with an atmospheric pressure gas chromatograph (Agilent Technologies) coupled to a Xevo TQS tandem mass spectrometer (Waters Corporation, Milford, Massachusetts, USA) operating in positive atmospheric-pressure chemical ionization. Quantification was based on isotope dilution using 13C-labeled internal standards. Procedural blanks, instrument blanks, in-house reference plasma, and NIST SRM 1957 were included in each analytical batch for quality assurance, and measured values were in good agreement with certified reference values.

We have used wet-weight PCB concentrations rather than lipid-normalized values as our main objective is to investigate how prenatal PCB exposure relates to metabolic and lipidomic profiles, including lipid-related pathways. Using lipid-normalized PCBs as the exposure could introduce bias, as the normalization denominator (total lipids) is closely related to the metabolic outcomes of interest and may act as a mediator rather than a pure confounder.

### 2.3 Target Analysis of Polar Metabolites by GC-QTOFMS

Maternal and cord plasma samples were randomized and prepared for GC–MS-based metabolomics analysis as previously described.^14^

Briefly, samples were extracted with methanol containing isotope-labeled internal standards, derivatized using methoxyamine hydrochloride (MOX) and MSTFA, and analyzed using GC–QTOF-MS. Quantification was based on external calibration curves that were generated from authentic standards. Quality control included pooled study samples, an NIST SRM 1950 serum, and in-house reference serum samples. Analytical reproducibility was within accepted limits, with average relative standard deviations of 12.3% in control samples.

### 2.4 Lipidomic Analyses by UHPLC-QTOFMS

Maternal and cord plasma samples were extracted using a modified Folch procedure as previously described.^15^ Samples were extracted with chloroform:methanol containing lipid-class-specific internal standards and analyzed using UHPLC-QTOFMS in positive electrospray ionization mode. Lipid identification was performed using an in-house spectral library, including retention time and MS/MS information. Quantification was based on internal standard normalization and lipid class-specific calibration curves. Pooled serum samples and control serum samples were included for quality control, and the analytical variation remained within accepted limits. Relative standard deviations (% RSDs) for peak areas for lipid standards representing each lipid class in the control serum samples (*n* = 12) and in the pooled serum samples (*n* = 77) were calculated on average 15.9% and 13.6% (raw variation) in maternal samples and in cord blood samples, respectively. The lipid concentrations in pooled control samples showed % RSDs within accepted analytical limits at averages of 14.7% and 20.4% for the maternal and cord blood serum samples, respectively.

### 2.5 Data Preprocessing

UHPLC-QTOFMS lipidomics data were processed independently from GC-QTOFMS metabolomics data. Mass spectrometry data was processed with MZmine 2.53 ^16^ as described in.^17^ Peak detection with a noise level of 1000 was performed first, followed with ADAP chromatogram builder including group intensity threshold to be as a noise level of (1000), minimum highest intensity of 10 and, an *m*/*z* tolerance of 0.009 *m*/*z* or 8 ppm. Next, chromatogram deconvolution was performed with local minimum search as algorithm with a 70% chromatographic threshold, 0.05 min of minimum retention time range, 5% minimum relative height, 2250 minimum absolute height, 1 as the minimum ratio of peak top/edge, and peak duration range in minutes from 0.08 to 5.00. Isotopic peak grouper was done next with an *m*/*z* tolerance of 0.05 *m*/*z* or 5 ppm, tR tolerance was set on 0.05 min, and a maximum charge of 2. For the alignment of peak lists, a Join alignment algorithm was performed with an *m*/*z* tolerance as 0.006 or 10.0 ppm and a weight of *m*/*z* as 2 with a tR tolerance of 0.1 and a weight of tR 1. Filtering with feature list rows’ filter was done next with three steps. First step rows that match with all criteria were kept with a retention time range from 2 to 12 and an *m*/*z* of 369–1200. Second filtering step removed rows that match with all criteria with a tR range of 2–4 and an *m*/*z* of 800–1200. Third filtering step removed rows that match with criteria with a tR range of 4–8 and an *m*/*z* of 370–500. Next, gap filling with peak finder was done with an *m*/*z* tolerance of 0.006 *m*/*z* or 10.0 ppm, a tR tolerance of 0.1 min, and with an intensity tolerance as 50%. Lastly, the identification with a custom database was done to identify the peak list with compound names.

### 2.6 Statistical Analysis

STATA 16.1 (StataCorp LLC) and MetaboAnalyst 6.0 ^18^ were used for statistical analyses. Prior to the data analysis, the three data sets were log2-transformed to approximate normal distribution and autoscaled/mean-centered and divided by the standard deviation of each variable. Values below the detection limit were imputed as half the limit of detection for each variable. To account for collinearity among highly correlated pollutant variables, principal component analysis (PCA) was applied, and the resulting principal component (PC) scores were used as exposure variables in the multivariable linear regression models. These models were used to assess associations between pollutant mixtures (PCs) and metabolites, lipids, and birth weight while adjusting for relevant covariates. In parallel, associations with individual POPs were evaluated using partial correlation analysis, controlling for the same covariates. This approach allowed an assessment of both mixture-level effects and compound-specific associations. For individual analysis of associations between POPs and metabolites/lipid classes, POPs with detection frequencies below 40% were excluded from the analysis. This criterion was applied to limit the impact of sparse data and nondetects, which can introduce bias and reduce the stability of partial correlation estimates. The chosen threshold represents a balance between retaining sufficient compounds for analysis and ensuring statistical robustness.

First, for the POP exposure data, Pearson pairwise correlations were investigated preliminarily to assess the relationships between the targeted 14 PCBs and 5 OC pesticides. Since *cis*- and *trans*-nonachlordane were detected in less than 10% of the samples (Figure 1), they were excluded from further statistical analyses.

Next, dimension reduction was performed by using PCA with an orthogonal varimax rotation for the investigation and identification of the main pollutant exposures underlying the observed variation in the data. The pollutant principal components (PCs) were selected based on eigenvalues of >1 and consequently five PC scores were generated from pollutant exposure loadings (Supporting Information Figure S1). Pollutant PC scores were treated as independent variables in subsequent regression analyses, while metabolites, lipids, and birth weight were modeled as dependent variables, with maternal age, prepregnancy BMI, and gestational age included as covariates where appropriate.

Two linear regression models were studied for the association between PC scores and lipids and metabolites. In model (1) multivariable linear regression modeled the metabolites and lipids on pollutant PCs adjusted for maternal age and model (2) modeled the metabolites and lipids on pollutant PCs with maternal age and prepregnancy body-mass-index (BMI). In both models, *p*-values were adjusted for multiple testing using the Benjamini–Hochberg procedure^19^ controlling the false discovery rate (FDR) at 10%. The covariates were selected based on previous studies where maternal age and prepregnancy BMI have been shown to influence exposure and/or birth weight. Age has been associated with differences in the cumulative POP exposure over time and with differences in placental transfer efficiency, which may influence both exposure and birth outcomes.^20,21^ Prepregnancy BMI has been shown to be associated with the body burden of the lipophilic POP exposure and may also influence birth weight through metabolic pathways.^22^ The covariates were assessed for multicollinearity, and residual plots were used to validate assumptions of linearity, normality, and homoscedasticity.

To investigate associations between prenatal POP exposure and birth weight, using maternal pollutant PCs, two models were studied. Model 1 was adjusted for maternal age, and model 2 was further adjusted for maternal prepregnancy BMI and gestational age, and *p* values were corrected for multiple testing using a 10% FDR. In addition, partial correlations, adjusted by age and BMI, were analyzed between individual POPs and metabolites, after log normalization and autoscaling. Maternal smoking during pregnancy and parity were evaluated as potential confounders, however, due to limited variability and/or missingness in these variables in our cohort, they were not included as confounders in the final models.

Correlation of POPs with dietary parameters and with individual POPs and metabolites was done using partial correlation analysis using MetaboAnalyst 6.0,^18^ with the data adjusted with maternal age and BMI for the maternal data, and additionally with infant sex, birth weight, and gestational age for the cord blood data.

Enrichment analysis was performed using MetaboAnalyst 6.0 applying both pathway-based and disease signature (blood) algorithms.^18^ In pathway-based enrichment, 3694 metabolite and lipid pathways from RaMP-DB (integrating KEGG via HMDB, Reactome, WikiPathways) were applied. For considering the pathway significantly altered, we have used *p* values (*p* <0.05) and the minimum number of significantly altered metabolites in a pathway ≥ 3. Also, LIpid Pathway Enrichment Analysis (LIPEA) analysis was applied (https://hyperlipea.org/home).

We also checked whether the birth weight and the z score were associated with metabolic profiles using partial correlation analysis adjusted with maternal age and BMI, gestational age, and infant sex. *Z* score measures how many standard deviations a newborn’s weight is from the mean for their gestational age and sex. The *Z* scores were calculated using two sex-specific references constructed from Nordic birth samples.^23^

## 3 Results

### 3.1 Maternal Exposure to PCBs and OC Pesticides and Their PCA Loadings

The EDIA cohort with characteristics of the participants and distribution of POPs in the mothers (*n* = 257) from the EDIA cohort are presented in Table 1. The mean age of participants was 31.7 years (18.4–45.8) and average BMI was 24.5 (±4.6). In total, 257 mothers had valid measurements of POPs with detection rates ranging from 8% to 100% depending on the analyte (Figure 2A). Among the studied PCBs, the highest detection frequencies and median concentrations were observed for PCB-153 (120.0 pg mL^–1^), PCB-138 (87.2 pg mL^–1^), and PCB-180 (57.6 pg mL^–1^) (Figure 2B). The lowest median concentrations were observed for congeners 189 (0.81 pg mL^–1^), 206 (0.71 pgmL^–1^), and 209 (0.82 pg mL^–1^). Among the studied OC pesticides, *p*,*p*’-DDE (97.14pg mL^–1^) and HCB (135.4 pg mL^–1^) were detected in >80% of the study participants while *cis*- and *trans*-chlordane were only detected in less than 10% of the subjects.

**Table 1:** Participant Characteristics and Distribution of Persistent Organic Pollutants in the Mothers (*n* = 257) from the EDIA Cohort^a^

characteristics	median (IQR) or percent (%)	
maternal age (years)	31.5 (18.5–45.8)	
pre-pregnancy BMI (kg m^–2^)	23.4 (16.9–45.7)	
gestational age (weeks)	40 (35.7–42.4)	
gender of the infant (%)	50/50	
infant birth weight (g)	3530 (1670–4720)	
maternal smoking status* (% nonsmokers/smokers)	94.5/5.5	
delivery type: vaginal/cesarean (%)*	87/13	
polychlorinated biphenyls (pg mL^–1^)	LOD	median (IQR)
PCB74	7.56	14.2 (11.1; 18.4)
PCB99	16.6	23.1 (19.2; 30.0)
PCB118	35.6	47.7 (40.0; 57.5)
PCB105	13.7	17.9 (15.6; 23.4)
PCB153	43.7	121.7 (90.8; 158.4)
PCB138	25.9	87.1 (67.8; 112.5)
PCB156	2.10	8.7 (6.4; 12.9)
PCB157	0.69	1.8 (0.9; 2.2)
PCB180	4.19	57.9 (38.8; 83.0)
PCB170	2.20	24.4 (16.1; 34.3)
PCB189	0.70	0.8 (0.4; 1.2)
PCB194	0.64	6.1 (3.2; 9.2)
PCB206	0.53	0.7 (0.8; 1.2)
PCB209	0.82	1.7 (1.4; 2.3)
organochlorine pesticides (pg mL^–1^)		
HCB	89.8	137.9 (118.8; 173.8)
Cis-Chlordane	16.6	21.8 (18.2; 28.6)
*Trans*-Chlordane	8.50	10.9 (9.7; 13.5)
*Trans*-Nonachlordane	2.70	9.2 (5.5; 15.7)
p,p’-DDE	5.40	99.7 (66.7; 153.0)

a Abbreviations: polychlorinated biphenyl (PCB), hexachlorobenzene (HCB), and dichlorodiphenyldichloroethylene (p,p’-DDE).

**Figure 2:**
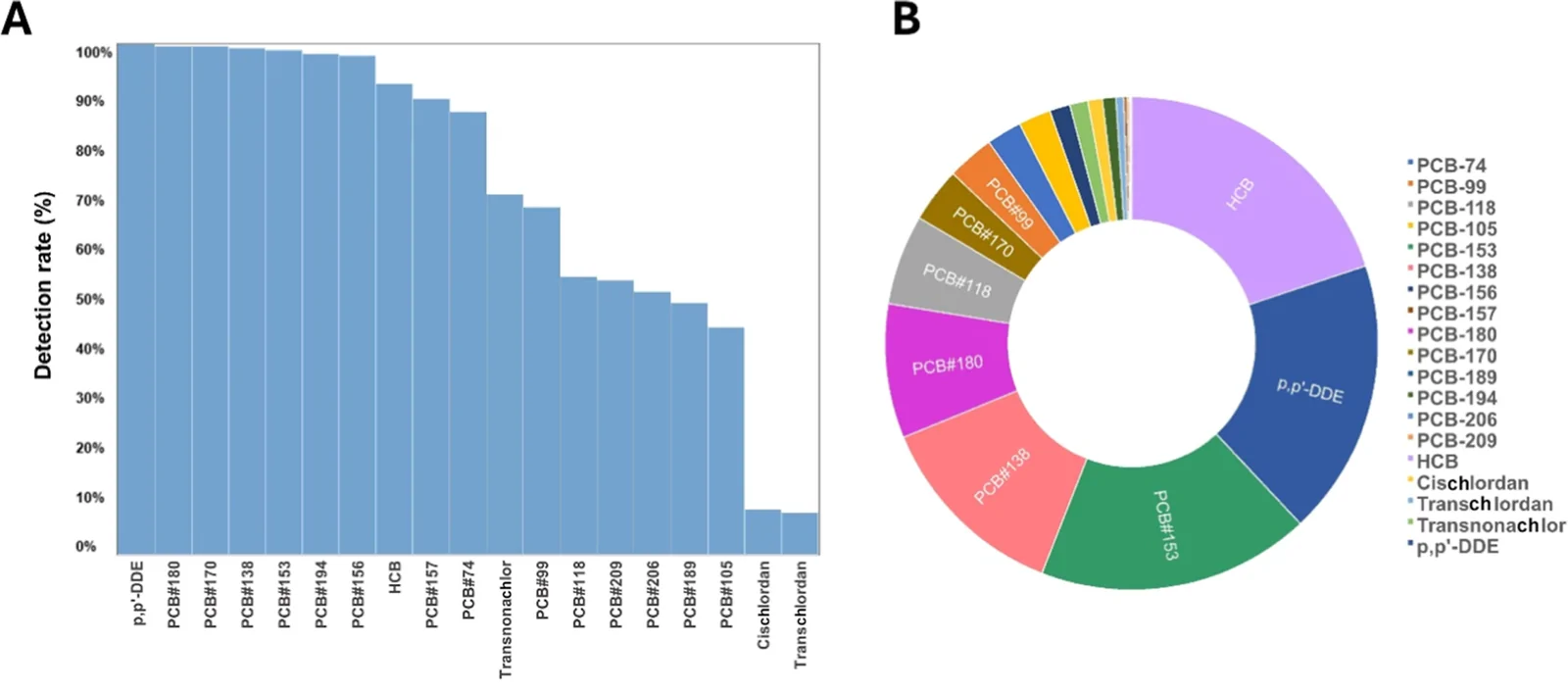
(A) Detection frequency of POPs in maternal plasma from the EDIA cohort. The detection rate (%) for each PCB and organochlorine pesticide. (B) Relative concentration profile of the cohort mean concentration for each compound, illustrating the relative contribution of individual POPs to the overall mean burden.

In Figure 3A, pairwise correlations between the PCBs and OC pesticides are displayed. The highest correlations (moderate) were observed between the PCBs 170, 180, 189, and 194 suggesting that these four PCBs not only share similar physicochemical properties but also originate from common sources. Moderate pairwise correlations between PCBs 156 and 157 as well as *cis*- and *trans*-chlordane were also observed. The correlations between remaining PCBs and OC pesticides were low suggesting that these markers of PCB and OC pesticide exposure are capturing different exposure dimensions, including their inherent environmental chemical properties, sources, trends, and their bioaccumulation potential throughout the food chains. Therefore, to capture this variability in the POP exposure data, PCA was performed and pollutant exposure PC loading scores were generated (Figure 3B). PC1 (21.4% of the variance explained) resulted in relatively high positive loadings for hepta- and decachlorinated PCBs 170, 180, 189, and 194. PC2 (10.2% of the variation explained) was highly loaded for penta- and hexachlorinated PCBs 99, 105, 118, and 138. PC3 (7.1% of the variation explained) was highly positively loaded for two PCBs 74 and 153 as well as the two readily detected OC pesticides *trans*-nonachlordane and *p*,*p*’-DDE. PC4 (6.4% of the variation explained) was positively highly loaded on PCBs 209, 206, 157, and 156. Finally, PC5 (6.1% of the variation explained) loaded highly primarily for HCB.

**Figure 3:**
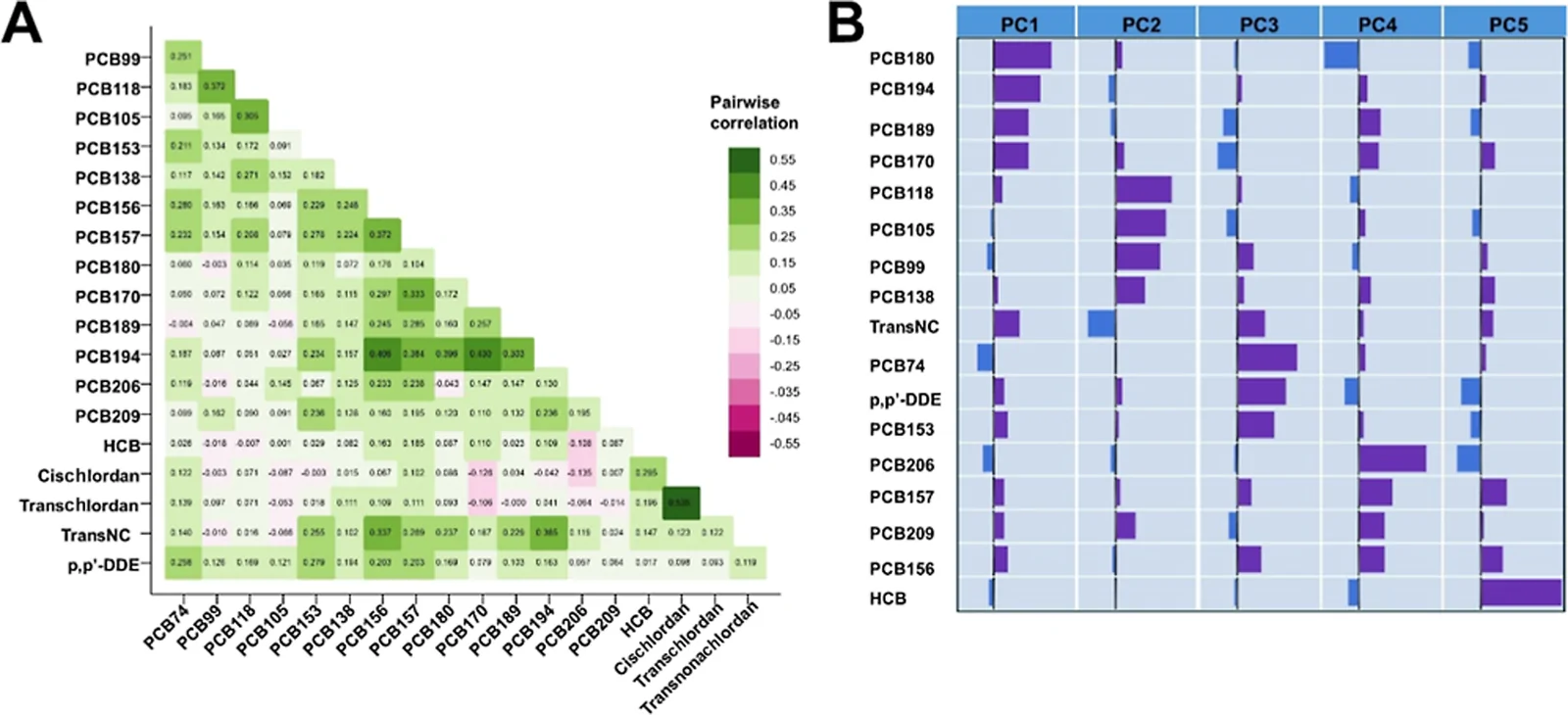
(A) Pairwise correlation coefficients between maternal PCBs and OC pesticide concentrations. (B) Loadings of individual POPs on the five principal components derived from maternal plasma concentrations. POP concentrations used in the PCA were pg mL^–1^ and natural log-transformed prior to analysis.

### 3.2 Dietary Sources of POP Exposure

The dietary pattern, in terms of product and quantity, might play a significant role in overall exposure in an individual. To better understand the specific sources of exposure in our study population, we therefore examined the relationship between PCBs and OC pesticides with maternal dietary profiles. Significant associations between specific dietary items and plasma concentrations of specific PCBs and OC pesticides were observed (Figure 4). Dietary item-specific analyses showed that the consumption of fish, shellfish, leafy vegetables, mushrooms, nuts, and seeds was positively correlated with several PCBs and OC pesticides. In contrast, consumption of poultry, lamb, oats and barley, coffee, pasta, and soft drinks showed inverse associations with PCB and OC pesticide levels. Maternal age was positively associated with several PCBs, whereas no significant associations were observed between POP concentrations and the maternal BMI.

**Figure 4:**
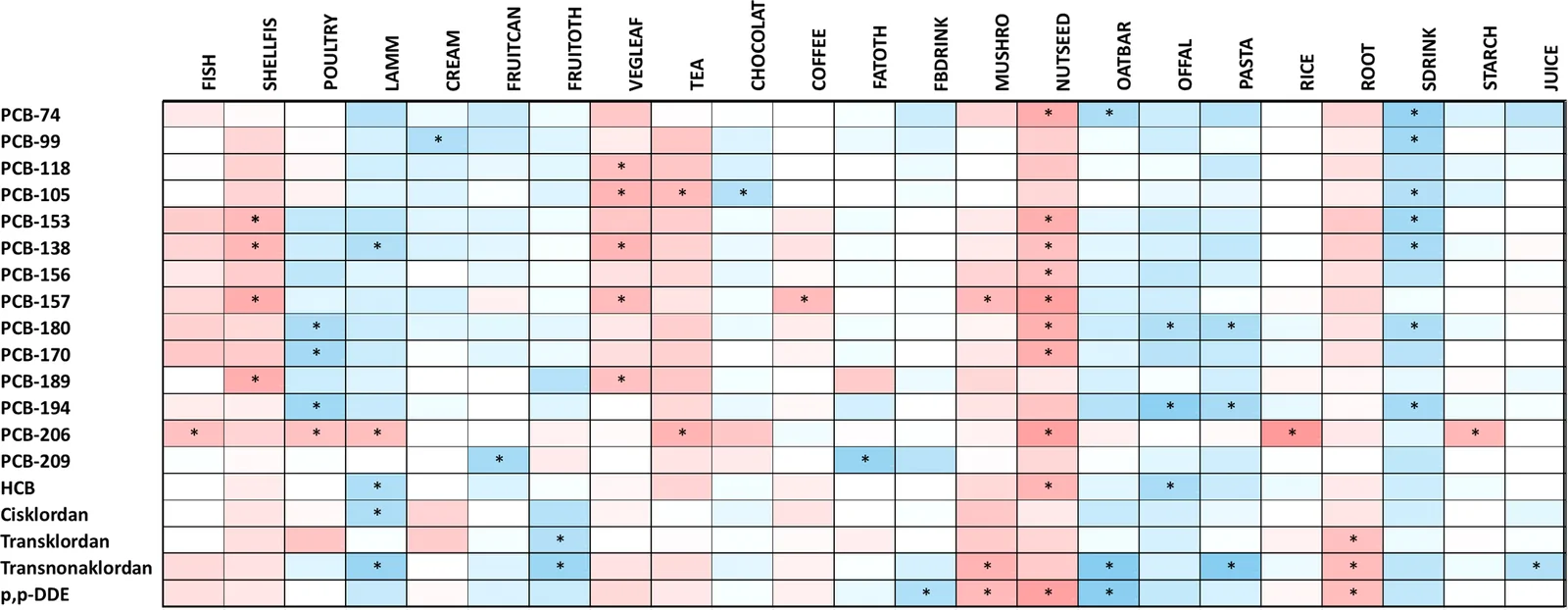
Heat map of Spearman’s correlation coefficients between maternal plasma concentrations of PCBs and OC pesticides with maternal dietary profiles. * Significant *p*-value <0.05.

### 3.3 Association of Maternal Metabolome with Maternal POP Exposure

Associations of five maternal POP PCs with both polar metabolites and lipids (full list of identified metabolites Supporting Information Table S1) were assessed. We found that PC3, which is mainly represented by PCBs 74 and 153 as well as *trans*-nonachlordane and *p*,*p*’-DDE, was found to be positively associated with lysine and octanoic acid following adjustment for maternal age, maternal BMI, and multiple correction using FDR < 0.10. Particularly the maternal age showed significant positive correlation with POP concentrations, while BMI showed associations mainly with the metabolome. A visualization of significant associations of pollutant exposure PC3 with metabolomics is displayed in Figure 5A.

**Figure 5:**
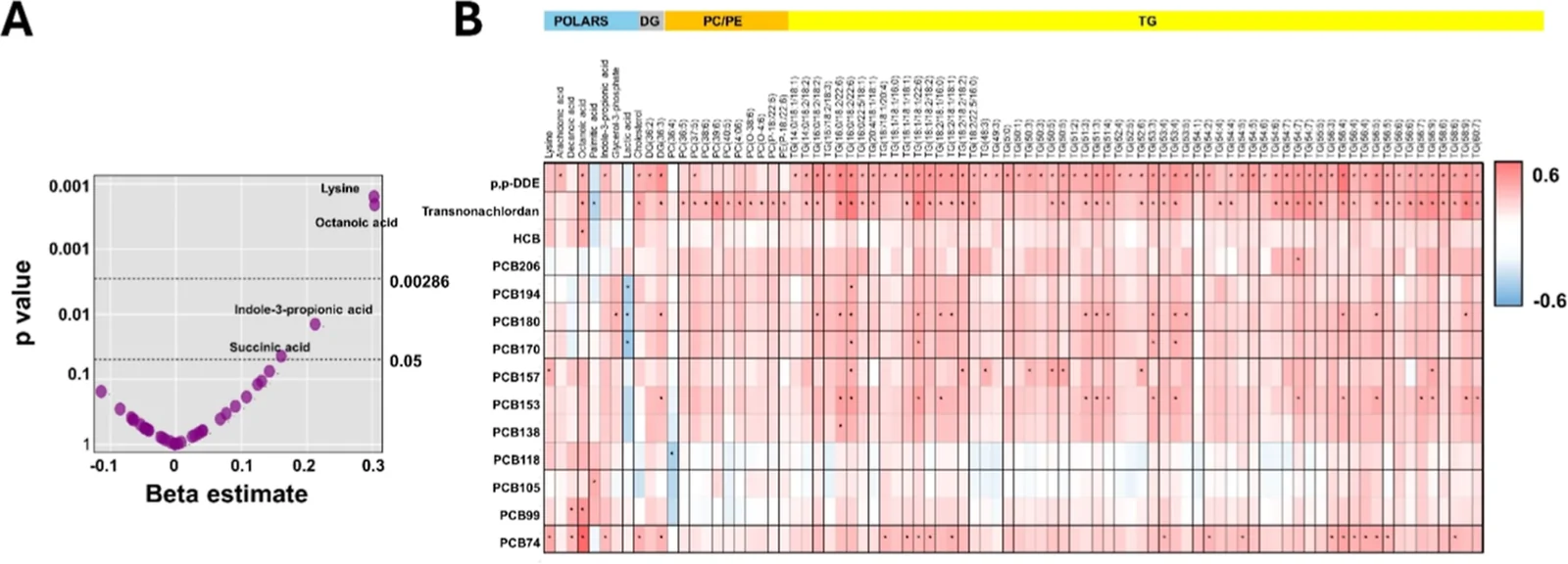
(A) Multivariable linear regression models for the associations between maternal pollutant PC3 (mainly represented by PCBs 74 and 153, *trans*-nonachlordane, and p,p’-DDE) with maternal metabolomic profiles after adjustment for maternal age and maternal prepregnancy BMI. (B) Partial correlations (adjusted by maternal age and BMI) between individual POPs (detection rate >40%) and metabolites, with only metabolites showing significant associations (* (*p* < 0.05, FDR <0.05)) with at least one of the POPs shown. Lipid abbreviations: DG = diacylglycerol; PC = phosphatidylcholine; PE = phosphatidylethanolamine; and TG = triacylglycerol. Fatty acid composition is indicated in parentheses as either the total carbon number and number of double bonds or as individual fatty acyls in the lipid. The prefixes ‘P’ and “O” denote alkyl ether (plasmalogen and alkyl ether) structures.

Associations of five maternal POP PCs and 268 lipids were assessed, and PC2, representing penta- and hexachlorinated PCBs, was found to be inversely associated with phosphatidylethanolamine PE (16:0/18:1) following adjustment for maternal age, maternal prepregnancy BMI, and multiple correction using FDR < 0.10. Next, we examined the associations of the five maternal POP PCs with composite measures of the 13 major lipid subclasses drawn from the 268 individual lipids measured. Results of the associations between each of the five pollutant PCs and the 13 lipid subclasses are shown in Table 2. Multiple associations of the five maternal pollutant PCs with lipid subclasses were observed in age as well as age and prepregnancy BMI-adjusted models. The most robust associations were observed for PC2, which was found to be inversely associated (*p* value = 0.001) with the PEs following adjustment for maternal age and prepregnancy BMI and multiple correction using FDR < 0.10. We observed positive associations of PC4 with polyunsaturated fatty acid triglycerides (TG_PUFA). PC5 was positively associated with diacylglycerols (DGs) and multiple correction using FDR < 0.10.

**Table 2:** Multivariable Linear Regression Models for the Associations between Maternal Pollutant Principal Components (PCs) and 13 Lipid Sub-Classes^a^

		model 1		model 2	
maternal pollutant PCs	lipid subclass	Beta (95% CI)	*p*-value	beta (95% CI)	*p*-value
PC1	Cer	0.04 (−0.005, 0.085)	0.086	0.038 (−0.009, 0.084)	0.112
	DG	0.047 (−0.02, 0.114)	0.171	0.051 (−0.018, 0.12)	0.151
	TG_PUFA	0.005 (−0.003, 0.013)	0.251	0.004 (−0.004, 0.013)	0.313
	TG_MUFA	0.002 (−0.002, 0.006)	0.257	0.002 (−0.002, 0.006)	0.416
	CE	–0.009 (−0.042, 0.024)	0.603	–0.012 (−0.045, 0.022)	0.501
	TG_SFA	0.002 (−0.006, 0.009)	0.633	0.002 (−0.006, 0.01)	0.634
	SM	–0.002 (−0.011, 0.007)	0.695	–0.002 (−0.011, 0.007)	0.657
	PC	–0.001 (−0.004, 0.003)	0.708	–0.001 (−0.004, 0.002)	0.658
	PE	–0.007 (−0.046, 0.032)	0.735	0 (−0.042, 0.041)	0.986
	PE_o/p	–0.009 (−0.061, 0.044)	0.749	–0.013 (−0.067, 0.041)	0.639
	LPC	–0.004 (−0.026, 0.019)	0.751	0 (−0.023, 0.023)	0.989
	PC_o/p	–0.001 (−0.008, 0.006)	0.780	–0.001 (−0.008, 0.006)	0.736
	PI	–0.008 (−0.184, 0.168)	0.929	0.03 (−0.152, 0.212)	0.749
PC2	PE	–0.063 (−0.097, −0.028)	0.001	–0.062 (−0.1, −0.025)	0.001
	Cer	–0.044 (−0.085, −0.003)	0.035	–0.045 (−0.088, −0.002)	0.040
	PE_o/p	–0.036 (−0.084, 0.011)	0.133	–0.035 (−0.085, 0.015)	0.172
	PC	–0.002 (−0.005, 0.001)	0.175	–0.002 (−0.005, 0.001)	0.216
	TG_PUFA	–0.005 (−0.012, 0.003)	0.233	–0.004 (−0.012, 0.004)	0.361
	PC_o/p	–0.004 (−0.01, 0.003)	0.276	–0.003 (−0.01, 0.004)	0.377
	DG	–0.032 (−0.093, 0.029)	0.306	–0.028 (−0.092, 0.036)	0.391
	SM	–0.004 (−0.012, 0.004)	0.367	–0.003 (−0.012, 0.005)	0.443
	LPC	–0.006 (−0.027, 0.014)	0.544	–0.007 (−0.028, 0.014)	0.527
	CE	–0.007 (−0.037, 0.023)	0.653	–0.006 (−0.038, 0.025)	0.688
	TG_MUFA	–0.001 (−0.004, 0.003)	0.697	0 (−0.004, 0.003)	0.910
	TG_SFA	–0.001 (−0.008, 0.006)	0.767	0 (−0.008, 0.007)	0.920
	PI	0.004 (−0.157, 0.164)	0.964	0 (−0.168, 0.169)	0.998
PC3	TG_PUFA	0.008 (0, 0.015)	0.054	0.008 (0, 0.016)	0.056
	TG_MUFA	0.003 (−0.001, 0.006)	0.101	0.003 (−0.001, 0.007)	0.123
	TG_SFA	0.005 (−0.002, 0.012)	0.134	0.005 (−0.002, 0.013)	0.136
	PE	–0.027 (−0.062, 0.009)	0.140	–0.026 (−0.064, 0.013)	0.192
	PI	–0.12 (−0.28, 0.04)	0.143	–0.098 (−0.267, 0.071)	0.257
	CE	–0.022 (−0.052, 0.008)	0.157	–0.022 (−0.053, 0.009)	0.167
	LPC	–0.014 (−0.035, 0.006)	0.164	–0.015 (−0.036, 0.007)	0.176
	DG	0.03 (−0.03, 0.091)	0.329	0.035 (−0.03, 0.099)	0.292
	Cer	–0.02 (−0.061, 0.021)	0.346	–0.02 (−0.063, 0.023)	0.359
	PE_o/p	–0.019 (−0.066, 0.029)	0.436	–0.018 (−0.069, 0.032)	0.475
	PC_o/p	–0.002 (−0.009, 0.004)	0.486	–0.002 (−0.009, 0.005)	0.523
	SM	–0.003 (−0.011, 0.006)	0.537	–0.003 (−0.011, 0.006)	0.551
	PC	–0.001 (−0.004, 0.002)	0.544	–0.001 (−0.004, 0.002)	0.563
PC4	TG_PUFA	0.008 (0.001, 0.015)	0.033	0.008 (0, 0.015)	0.038
	PI	–0.137 (−0.287, 0.013)	0.074	–0.098 (−0.252, 0.056)	0.215
	TG_MUFA	0.002 (−0.001, 0.006)	0.152	0.002 (−0.001, 0.005)	0.241
	PE	–0.019 (−0.052, 0.015)	0.271	–0.011 (−0.046, 0.024)	0.544
	TG_SFA	0.003 (−0.003, 0.01)	0.302	0.003 (−0.003, 0.01)	0.350
	DG	0.027 (−0.03, 0.085)	0.350	0.041 (−0.018, 0.1)	0.175
	PE_o/p	–0.018 (−0.063, 0.027)	0.433	–0.013 (−0.059, 0.034)	0.593
	CE	–0.011 (−0.039, 0.017)	0.436	–0.012 (−0.041, 0.016)	0.405
	LPC	–0.005 (−0.025, 0.014)	0.577	–0.003 (−0.022, 0.017)	0.794
	SM	–0.001 (−0.009, 0.006)	0.746	–0.001 (−0.009, 0.007)	0.780
	Cer	–0.005 (−0.043, 0.034)	0.818	0.001 (−0.038, 0.041)	0.945
	PC	0 (−0.003, 0.002)	0.829	0 (−0.003, 0.003)	0.889
	PC_o/p	0 (−0.006, 0.006)	0.976	0 (−0.006, 0.006)	0.985
PC5	DG	0.061 (0.009, 0.112)	0.022	0.07 (0.016, 0.124)	0.012
	TG_MUFA	0.002 (−0.001, 0.005)	0.107	0.002 (−0.001, 0.006)	0.115
	CE	–0.018 (−0.043, 0.008)	0.172	–0.019 (−0.046, 0.007)	0.155
	TG_PUFA	0.004 (−0.003, 0.01)	0.253	0.004 (−0.003, 0.011)	0.265
	PE_o/p	–0.018 (−0.059, 0.022)	0.381	–0.018 (−0.061, 0.025)	0.406
	PC_o/p	–0.002 (−0.008, 0.003)	0.416	–0.002 (−0.008, 0.003)	0.402
	SM	–0.002 (−0.009, 0.004)	0.480	–0.003 (−0.01, 0.004)	0.458
	TG_SFA	0.002 (−0.004, 0.008)	0.551	0.002 (−0.004, 0.008)	0.518
	LPC	0.004 (−0.013, 0.022)	0.617	0.003 (−0.015, 0.021)	0.753
	PC	0 (−0.003, 0.002)	0.691	–0.001 (−0.003, 0.002)	0.659
	PI	–0.023 (−0.16, 0.114)	0.742	–0.032 (−0.175, 0.111)	0.664
	Cer	0.005 (−0.03, 0.04)	0.776	0.009 (−0.028, 0.045)	0.641
	PE	0.004 (−0.027, 0.034)	0.813	0.008 (−0.025, 0.04)	0.644

a “Model 1” shows associations following adjustment for age. “Model 2” shows associations following adjustment for age and pre-pregnancy BMI.

We also analyzed associations between individual POPs with detection rate >40% and metabolites (Figure 5B) using partial correlation after adjustment with age and prepregnancy BMI. *p*,*p*′-DDE and *trans*-nonachlordane were showing stronger associations than PCBs, being positively associated with large number of TGs, multiple DGs, and polyunsaturated fatty acyl containing PCs. *p*,*p*′-DDE and PCB-74 showed also positive association with gut microbiota-derived indole-3-propionic acid. PCBs were showing positive association mainly with TGs and some DGs, adverse association with free cholesterol, with PCBs 74, 163, 170, and 180 showing most associations with individual lipids. The majority of the lipids showing positive associations were containing PUFAs or monounsaturated fatty acids (MUFAs).

Enrichment analysis was performed using the data for those individual POPs showing strongest association with metabolome because analyses based on PCB clusters resulted in only a limited number of significantly altered metabolites and lipids. This reduced number was not sufficient to support robust and reliable pathway enrichment analysis. As majority of the metabolic changes were in the lipids, we also performed LIPEA analysis. For *trans*-nonachlordane, the following pathways were enriched (p, FDR < 0.05, minimum 3 significant metabolites): retinoid metabolism and transport, metabolism of fat-soluble vitamins, and peptide hormone metabolism. For PCB-153 and *p*,*p*′-DDE, similar pathways were enriched, but the number of significant metabolites was under 3, and thus, the results are not sufficiently robust. Interestingly, for PCB-153 and *trans*-nonachlordane, the disease pathways affected included obesity and pregnancy, and additionally schizophrenia for *p*,*p*′-DDE and *trans*-nonachlordane. LIPEA pathway analysis showed that pathways related to fat digestion and absorption, cholesterol metabolism, and vitamin digestion and absorption were altered, particularly for *trans*-nonachlordane.

### 3.4 Associations of Gestational POP Exposure, Birth Weight, and Cord Blood Metabolome

Associations of prenatal pollutant exposure PCs and neonatal birth weight were assessed by using multivariable linear regression. In Table 3, the associations between the pollutant exposure PCs and birth weight are presented. “Model 1” was adjusted for maternal age, while “model 2” was adjusted for maternal age, maternal prepregnancy BMI, and gestational age. *p*-values were adjusted for multiple correction using FDR < 0.10. None of the pollutant exposure PCs were significantly (*p* < 0.05) associated with birth weight in the two models. However, when considering effect modification by sex in the regression models, we found that prenatal pollutant exposure PC1 was inversely associated with birth weight among girls (nominal *p* = 0.037) after adjustment for maternal age but that the association attenuated after multiple adjustment (*p* = 0.074) (Supporting Information Figure S2). There was no significant effect modification by sex for the remaining pollutant exposure PCs with birth weight. We also checked whether the birth weight was associated with metabolic profiles and found that three phospholipids (LPC(20:3), PC(40:6), and PC(40:8)) and one TG (TG­(18:0/18:1/20:4)) showed inverse association (*R* −0.2, *p* < 0.001, FDR <0.1) with the Z score, after adjustment with maternal age and BMI, gestational age, and infant sex.

**Table 3:** Associations of Prenatal Pollutant PCs with Neonatal Birth Weight in “Model 1” Adjusted with Maternal Age and “Model 2” Adjusted for Maternal Age, Gestational Age, and Maternal Pre-pregnancy BMI

pollutant exposure PCs	birth weight model 1		birth weight model 2	
	Β (95% CI)	*p*-value	Β (95% CI)	*p*-value
PC1	–45.3 (−82.47, −8.31)	0.017	–25.19 (−60.9, 10.50)	0.168
PC2	–4.31 (−47.59, 38.96)	0.845	5.69 (−33.06, 44.45)	0.774
PC3	–1.96 (−45.37, 41.43)	0.929	5.27 (−33.29, 43.84)	0.789
PC4	–37.49 (−81.65, 6.67)	0.097	–25.01 (−67.14, 17.11)	0.246
PC5	–41.03 (−92.63, 10.5)	0.120	–40.19 (−85.55, 5.15)	0.084

Prenatal PCB exposure was associated with alteration of metabolome, particularly related with lipid signature (Figure 6, Supporting Information Table S2). At the lipid class level, several PCBs (74, 99, 105, 118, 138, 153, 157, and 170) showed inverse association with most lipid classes, with cholesterol esters not being associated with any of the PCBs. At the individual metabolite level, a similar negative association was observed, especially for TGs and multiple phospholipids containing PUFAs. Additionally, three TGs showed positive association with the PCBs. At the disease pathway level, PCB-99 showed an association with obesity, pregnancy, celiac disease, and very long chain acyl-CoA dehydrogenase deficiency (*p*, FDR < 0.05, >3 significant metabolites in each pathway). Also, PCB-118 was associated with obesity and pregnancy outcomes.

**Figure 6:**
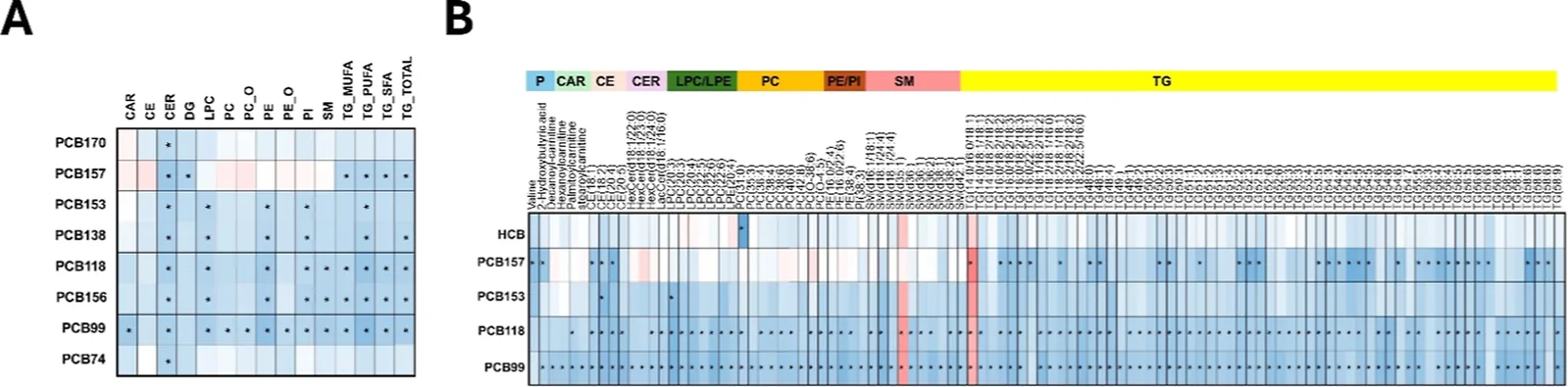
Partial correlations between prenatal POP levels and cord blood metabolome in (A) lipid class level and (B) on the level of individual metabolites. Only compounds showing significant association between metabolites and at least a single POP are shown. Significant associations are marked with * (*p* < 0.05, FDR < 0.1). Lipid abbreviations: CAR = carnitines, CE = cholesterol ester, CER = ceramides DG = diacylglycerol, (L) PC = (lyso) phosphatidylcholine, (L) PE = (lyso)­phosphatidylethanolamine, *P* = polar metabolites, PI = phosphatidylinositol, SM = sphingomyelin, and TG = triacylglycerol (MUFA = monounsaturated fatty acids, PUFA = Polyunsaturated fatty acids, SFA = saturated fatty acids). Fatty acid composition is indicated in parentheses as either the total carbon number and number of double bonds or as individual fatty acyls in the lipid. The prefixes “*P*” and “*O*” denote alkyl ether (plasmalogen and alkyl ether) structures.

We also assessed the correlations between the maternal metabolome and the cord blood metabolome (Supporting Information Table S3). Most correlations were relatively weak (|*R*| < 0.3), with the strongest observed between maternal and cord blood threonine (*R* = 0.40, *p* = 2.25 × 10^–10^).

A clear positive correlation was also observed between maternal ceramides (Cer (d18:1/24:0) and Cer (d18:1/24:1)) and DG (34:1) and cord blood ceramides and phospholipids. These metabolites also showed positive correlations with a large number of triacylglycerols (TGs). Overall, TGs showed very low correlations between maternal and cord blood samples, and a similar pattern was observed for amino acids and free fatty acids. The majority of maternal phospholipids showed positive correlations with cord blood phospholipids; however, these correlations were generally weak (|*R*| < 0.3).

## 4 Discussion

A broad range of targeted PCBs and organochlorine (OC) pesticides were detected in the EDIA cohort, with concentrations spanning several orders of magnitude. The high detection frequencies and wide concentration ranges observed in this maternal cohort reflect the long-term persistence and bioaccumulative nature of these compounds. When compared with other mother–child cohort studies, maternal serum concentrations in our study were on similar levels to those earlier reported in European populations.^24–28^ Overall, PCB concentration patterns aligned with previous studies for most congeners. However, PCB118 accounted for a markedly higher share of total PCBs (23% vs 3%–6% in earlier reports), while PCB170 and PCB180 contributed substantially less (1.4% and 3% vs 23%–26% in other studies).^27,29–31^

Assessment of exposure levels, together with identification of potential exposure sources, is important for evaluating possible health risks in the general population. From this perspective, investigating the relationship between plasma PCB and OC pesticide concentrations and specific dietary items may identify and highlight specific food items that are important for these exposures. Our study indicated that self-reported consumption of specific dietary items, such as shellfish, nuts, and seeds, were positively correlated with several PCBs and OC pesticides, thus suggesting that these dietary items to be a possible source of POPs. On the other hand, consumption of poultry, lamb, oat and barley cereals, coffee, pasta, and soft drinks, showed an inverse association with plasma concentrations of PCBs and OC pesticides. It should be noted that the observed associations with nuts and vegetables may reflect indirect dietary patterns, coconsumption behaviors, or residual confounding rather than these foods being primary sources of POP exposure. Our results agree with previous studies reporting that food items with a high fat content such as fish and shellfish as well as dairy products are of particular concern.^32–34^ While seafood is a known source of many POPs due to bioaccumulation, the positive association with nuts and seeds is probably due to the contamination at some part of the food/production chain. The correlation between fish intake and PCB levels was not strong, despite previous reports from Finland showing such associations.^35^ However, the dietary data reflect intake during pregnancy, which may not capture prepregnancy fish consumption. This is particularly relevant given dietary recommendations during pregnancy that may lead to reduced fish intake.

We also observed associations between maternal POP exposure and maternal metabolic profiles. The strongest associations were observed for maternal pollutant exposure PC2 scores with PEs as well as for maternal pollutant exposure PC3 scores with the amino acid lysine and the medium-chain saturated fatty acid octanoic acid. In PC3, looking at the individual compounds driving the associations, showed significant association with PCB74 and near-significant association with *p*,*p*′-DDE (FDR = 0.053), suggesting that these metabolites may reflect both the influence of individual compounds and the combined effect of coexposure to correlated POPs. This indicates that the mixture pattern captured by PC3 may enhance detection of biologically relevant associations that are weaker when individual compounds are considered separately. In PC3, the grouping of PCB 74 and PCB 153 with *trans*-nonachlordane and *p*,*p*′-DDE within the same principal component likely reflects correlated internal exposure patterns, potentially driven by their shared persistence, lipophilicity, bioaccumulation, and partly overlapping environmental or dietary exposure sources., Among individual POPs, the majority of the associations were with MUFA- and PUFA-containing TGs and PCs. The observed increase in TGs following PCB exposure may be related to enhanced triglyceride mobilization driven by increased energy requirements.^36^ Overall, PCB exposure has been linked with elevated serum concentrations of lipids.^37–39^ Several other studies have also investigated associations of exposure to various environmental contaminants including perfluoroalkyl substances, phthalates, heavy metals, and air pollution with maternal metabolomic profiles. However, we are aware of only three studies investigating the associations of maternal pregnancy exposure levels to POPs, mainly PCBs and OC pesticides, with circulating metabolomic and lipidomic profiles. In a study including 93 women from the Chiba Study of Mother and Children’s Health (C-MACH) cohort from Japan, associations of maternal PCB concentrations with serum metabolomic profiles were studied.^40^ Five metabolites representing mainly glutathione and amino acid metabolism were associated with high (quartile 4) PCB concentrations, however, these models did not address any covariates, and the methodology did not cover lipids. Hu et al. assessed nontargeted metabolomics features of maternal exposure to *p*,*p*′-DDT, *p*,*p*′-DDE, and *o*,*p*′-DDE in 397 mothers from the Child Health and Development Studies (CHDS) in samples collected in California between 1959 and 1967 and observed a cluster of pathways, including fatty acid metabolism, to be associated with *p*,*p*′-DDE exposure after adjustment for multiple comparisons.^41^ The metabolic platform was limited in respect of neutral lipids such as DGs and TGs. The findings linking exposure to DDT with fatty acid metabolism have also been reproduced in an experimental study where adult mice perinatally exposed to DDT caused dysregulation of fatty acid and oxidative respiration metabolism, possibly reflecting the impacts of the persistent DDE metabolites.^42^ Partly in line with these studies and despite the apparent methodological differences in study design, sampling time, exposure burdens, and metabolomics coverage, we also observed that higher maternal pollutant exposure was associated with concentrations of several lipid classes (TG, PE, PC, DG), the saturated fatty acid octanoic acid, and the amino acid lysine. In addition, *p*,*p*′-DDE and PCB-74 showed positive association with the gut microbiota-derived metabolite indole-3-propionic acid. Indole-3-propionic acid is a gut microbiota-derived tryptophan metabolite that reaches the circulation and contributes to epithelial barrier and immune homeostasis via xenobiotic sensing pathways,^43^ suggesting potential alteration of the gut microbiota due to exposure, as have been also implicated in other studies.^44^ Maternal blood triglyceride levels have been associated with gestational complications, especially late-term elevated levels.^45,46^ The enrichment analysis for top three POPs showing association with the metabolome indicated that the association were linked with obesity and pregnancy, with pathways related to fat digestion and absorption as well as vitamin digestion, absorption, and metabolism. Altered vitamin digestion and absorption during pregnancy can have several implications for both maternal health as well as for the developing fetus.

Another subsequent effect of maternal metabolomic and lipidomic alterations in response to POP exposure is potential alteration of maternal-fetal nutrient transfer, with implications for fetal development and anthropometric measures.^47,48^ From this perspective, prenatal exposure to POPs has been previously linked with reduced birth weight, however, inconsistent results have been reported, where, e.g., single pollutants were inversely associated with birth weight while other pollutants were positively associated and there are also studies in which no effects were observed.^7,8,49^ Earlier studies have also highlighted that POPs may influence birth weight in a sex-specific way,^8^ since endocrine-disrupting chemicals may exert sexually dimorphic effects, with implications for energy homeostasis, xenobiotic metabolism, and glucose and lipid metabolism.^50^ Sex-specific associations are biologically plausible because the placenta has been reported to exhibit intrinsic sexual dimorphism in endocrine signaling and adaptive responses to environmental stressors, which can differentially influence placental function and fetal growth trajectories.^51^ Consistent with this, a study from a large pooled European birth-cohort including 9000 mother–child pairs have reported effect modification by fetal sex for prenatal PCB-153 and *p*,*p*′-DDE exposure and fetal growth outcomes and reported stronger adverse growth signals in girls.^52^ We observed weak to no associations of prenatal pollutant exposure PC scores with neonatal birth weight at baseline. However, when we stratified our analyses by sex, an inverse association of PC1 (showing positive loadings for hepta- and decachlorinated PCBs 170, 180, 189, and 194) and birth weight was observed among the girls in our study. This association lost significance after performing multiple adjustments. The lack of strong associations between the PCB concentrations with birth weight in our study population may be because of the general decline in POP exposure following regulatory restrictions. Several earlier studies reporting associations with birth weight were conducted in populations with higher exposure levels, where effects may be more readily detectable.

Although the association with birth weight was only modest, exposure to POPs was linked to significant changes in lipid profiles in the offspring, particularly in the cord blood. The cord plasma lipidome reflects various aspects of metabolism, including maternal, placental, and fetal processes, as well as the transfer of nutrients across the maternal-placental-fetal axis. It provides valuable information regarding fetal nutrient availability, metabolic status, and the presence of bioactive lipid signaling molecules in fetal circulation. Notably, the generally weak correlations observed between maternal and cord blood metabolomes suggest that fetal metabolic profiles are not merely a direct reflection of maternal metabolism but rather are shaped by selective transfer and independent fetal and placental regulation. Most lipids exhibited negative associations with POP exposure, with TGs and several PUFA-containing lipids showing particularly strong negative correlations. Transplacental delivery of fatty acids and cholesterol is actively regulated by placental transport/binding proteins and cholesterol transporters, which shape maternal-fetal lipid gradients during gestation.^53^ The combination of reduced PUFA levels in cord blood and increased PUFA-containing lipids in maternal circulation may reflect impaired transplacental lipid transfer and altered fetal–placental lipid metabolism. This is particularly relevant because the fetus is largely dependent on maternal supply of long-chain PUFAs due to limited endogenous desaturase activity. This can potentially have an impact of the fetal and infant health, as PUFAs are essential fatty acids with vital physiological functions, playing a key role in brain development during fetal, neonatal, and early childhood stages. Although POPs show compound specific maternal-fetal partitioning, it is unclear whether POP exposure alters specific lipid transporter function.^54^ Although most maternal–cord blood correlations were weak, the consistent positive associations observed for phospholipids and certain ceramides suggest some degree of coordinated lipid metabolism or selective transfer, whereas other metabolite classes such as TGs and amino acids appear to be more independently regulated.

We acknowledge our study has limitations. While the sample size was reasonable, it remains relatively small. Additionally, the concentrations of POPs were low, which may limit the generalizability of our findings, particularly since the cohort was geographically restricted to a specific region, with ethnically and socioeconomically uniform population. Also, because parity data were not collected and smoking prevalence was low (∼5%), we could not robustly adjust for these factors. However, the strengths of the study include the well-characterized cohort, matched mother-infant samples, inclusion of dietary data during pregnancy, and comprehensive coverage of the metabolome.

## 5 Conclusions

Taken together, our study shows that although the concentrations of persistent organochlorine pollutants have decreased in the environment and in humans over the past few decades, trace levels can still be detected. We found that higher maternal persistent organic pollutants were associated with several metabolomic and lipidomic measures in both mothers and infants at birth. Our findings underscore the importance of investigating maternal environmental exposures and provide novel insights into potential mechanisms underlying maternal exposure to persistent organic pollutants, with potential implications for maternal health and pregnancy outcomes and their potential effects on neonatal outcomes.

## Supplementary Material





## Data Availability

Data are available upon request and an appropriate institutional collaboration agreement. These data are not available to access in a repository owing to concern that the identity of participants might be revealed inadvertently.

## Notes

Scree plot of eigenvalues from PCA of pollutant exposure data, relationship of maternal pollutant PC1 with neonatal birth weight, marginal probabilities of maternal pollutant PC1 score for birth weight, and identified metabolites and the measurement type (PDF)
XLSX
