# Supplementary material for: Prenatal Exposure to Persistent Organic Pollutants Is Associated with Altered Lipid and Metabolomic Profiles in Mothers and Newborns
Source: Environ Health (Wash). 2026 Jun 9;4(9):2106–18. doi: 10.1021/envhealth.6c00104 (PMC13595375; doi:10.1021/envhealth.6c00104)
Supplement: Supplementary file 1 [file eh6c00104_si_001.pdf]

Supporting Information

Prenatal exposure to persistent organic pollutants is associated with altered lipid and metabolomic profiles in mothers and newborns

Samira Salihovic, Tim Sinoja, Suvi M. Virtanen, Matej Orešič, Mikael Knip, Tuulia Hyötyläinen*

Content

- Supplementary Figure 1
- Supplementary Figure 2
- Supplementary Table 1
- Supplementary Tables 2 and 3 are provided as Excel files

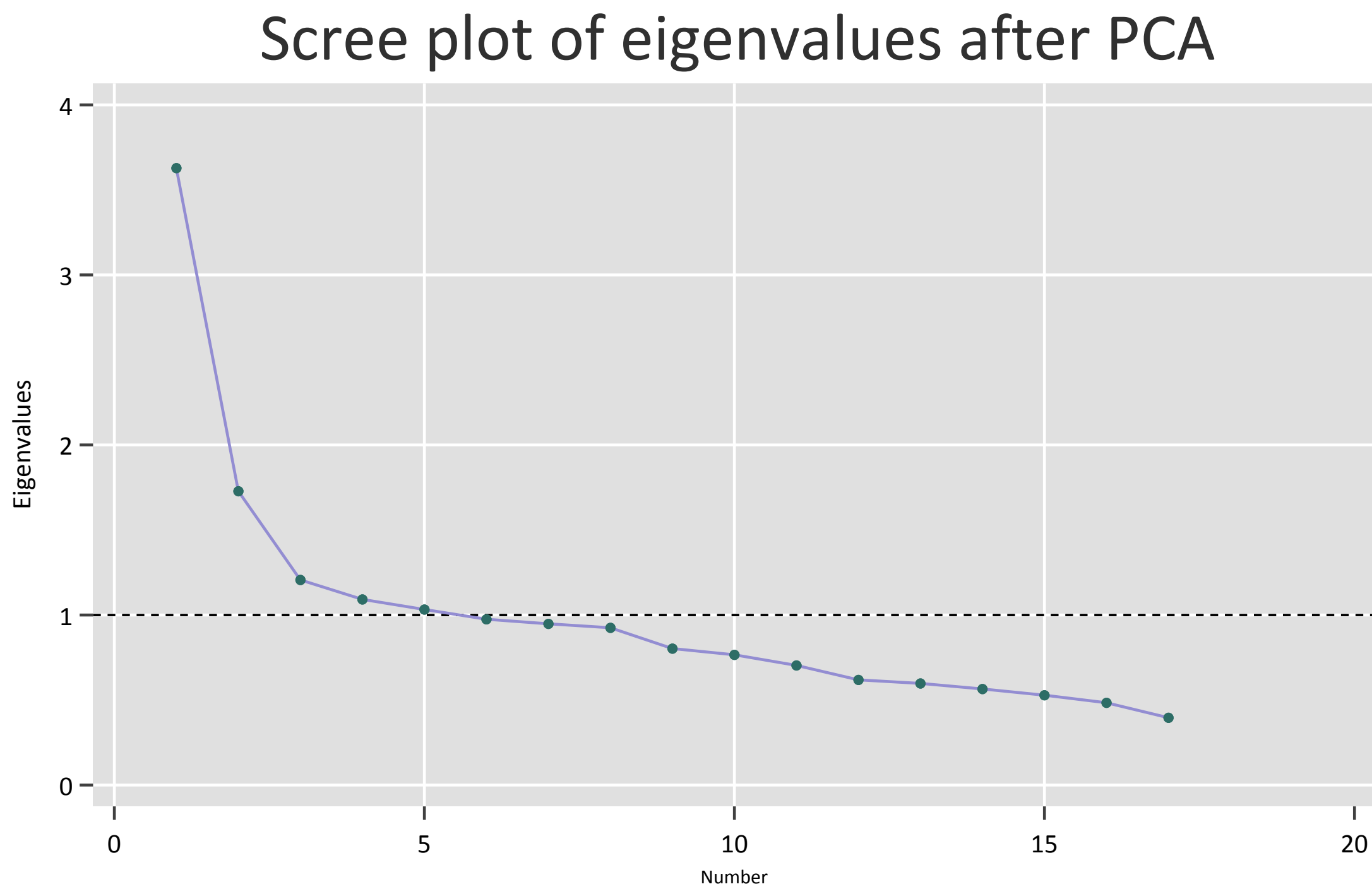

Supplementary Figure 1. Scree plot of eigenvalues from PCA of pollutant exposure data. The scree plot displays the eigenvalues (variance explained) for each principal component derived from the pollutant exposure matrix, ordered from PC1 onward. PCs with eigenvalue >1 were used in the present study.

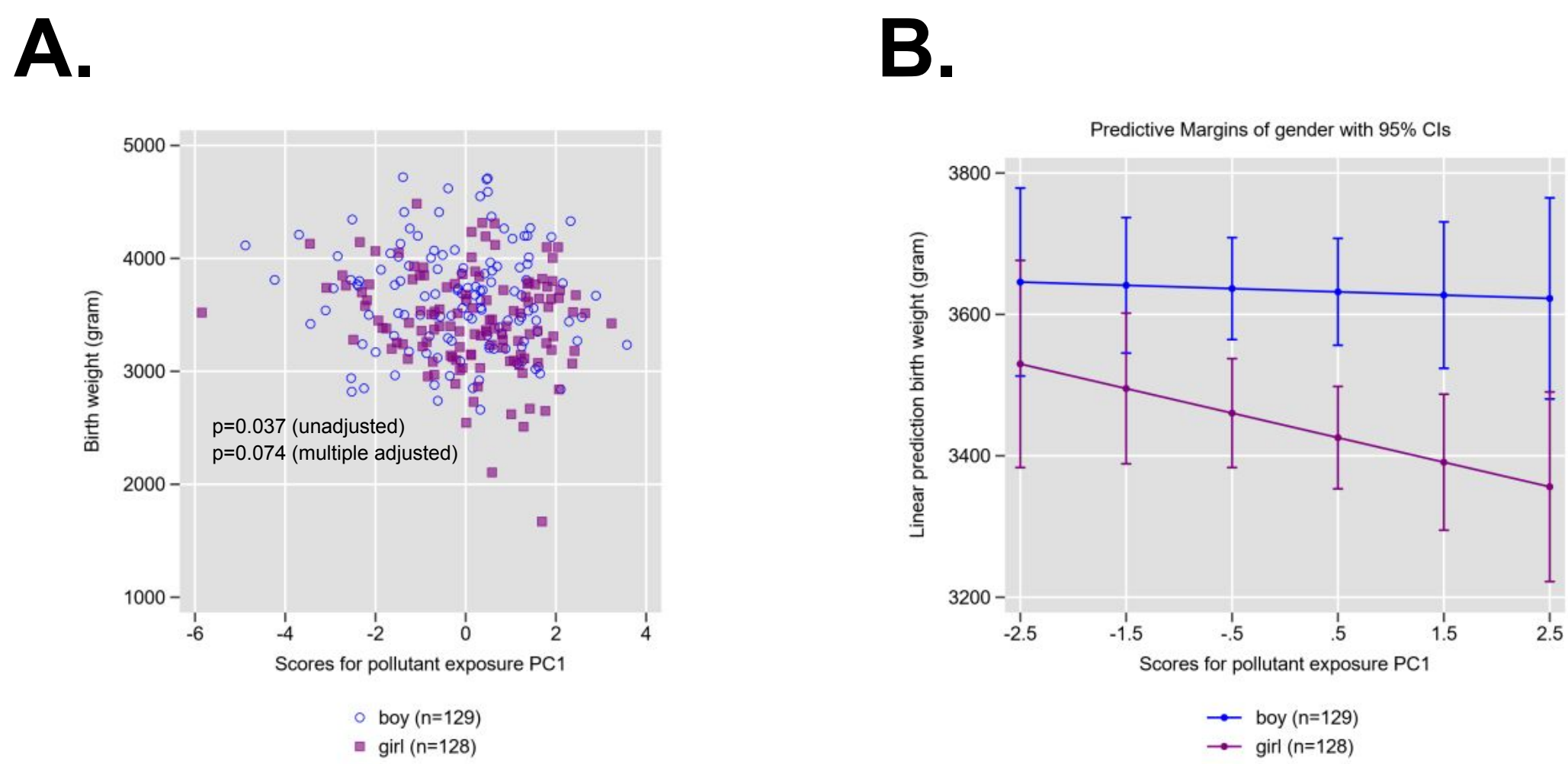

Supplementary Figure 2. (**A**) Relationship of maternal pollutant PC1 with neonatal birthweight among boys (blue dots) and girls (purple dots) and (**B**) marginal probabilities of maternal pollutant PC1 score for birth weight among boys (blue line) and girls (purple line).

Supplementary Table 1. Identified metabolites, their level of identification based on Metabolomics standard initiative, and the measurement type. The metabolites measured with GC-QTOFMS platform are targeted, quantitative measurements while lipidomics is semi-targeted and semi-quantitative. Lipid abbreviations: CAR= carnitines, CE= cholesterol ester, CER= ceramides DG = diacylglycerol; (L)PC = (lyso) phosphatidylcholine; (L) PE = (lyso)phosphatidylethanolamine; P= polar metabolites, PI= phosphatidylinositol, SM= sphingomyelin, TG = triacylglycerol (MUFA= monounstaryrated fatty acids, PUFA? Polyunsaturated fatty acids, SFA= saturated fatty acids). Fatty acid composition is indicated in parentheses as either the total carbon number and number of double bonds, or as individual fatty acyls in the lipid. The prefixes 'P' and 'O' denote alkyl ether (plasmalogen and alkyl ether) structures. GC= gas chromatography, UHPLC= ultra-high performance liquid chromatography, QTOFMS = quadrupole-time of flight mass spectrometry.

Metabolite	ID level	Platform
Lactic acid	1	GC-QTOFMS
Alanine	1	GC-QTOFMS
2-Hydroxybutyric acid	1	GC-QTOFMS
3-Hydroxybutyric acid	1	GC-QTOFMS
Valine	1	GC-QTOFMS
Octanoic acid	1	GC-QTOFMS
Leucine	1	GC-QTOFMS
Isoleucine	1	GC-QTOFMS
Proline	1	GC-QTOFMS
Glycine	1	GC-QTOFMS
Succinic acid	1	GC-QTOFMS
Fumaric acid	1	GC-QTOFMS
Serine	1	GC-QTOFMS
Threonine	1	GC-QTOFMS
Decanoic acid	1	GC-QTOFMS
Methionine	1	GC-QTOFMS
Aspartic acid	1	GC-QTOFMS
Glutamic acid	1	GC-QTOFMS
Phenylalanine	1	GC-QTOFMS
Glycerol-3-phosphate	1	GC-QTOFMS
Glutamine	1	GC-QTOFMS
Ornithine	1	GC-QTOFMS
Citric acid	1	GC-QTOFMS
Ornithine	1	GC-QTOFMS
Lysine	1	GC-QTOFMS
Fructose	1	GC-QTOFMS

Tyrosine	1	GC-QTOFMS
Palmitic acid	1	GC-QTOFMS
Indole-3-propionic acid	1	GC-QTOFMS
Linoleic acid	1	GC-QTOFMS
Oleic acid	1	GC-QTOFMS
Stearic acid	1	GC-QTOFMS
Arachidonic acid	1	GC-QTOFMS
Cholesterol	1	GC-QTOFMS
CE(18:1)	1	UHPLC-QTOFMS
CE(16:0)	1	UHPLC-QTOFMS
CE(20:4)	2	UHPLC-QTOFMS
CE(20:5)	2	UHPLC-QTOFMS
Cer(d18:1/23:0)	2	UHPLC-QTOFMS
Cer(d18:1/24:0)	1	UHPLC-QTOFMS
Cer(d18:1/24:1)	2	UHPLC-QTOFMS
DG(34:1)	2	UHPLC-QTOFMS
DG(36:2)	2	UHPLC-QTOFMS
DG(36:3)	2	UHPLC-QTOFMS
LPC(16:0e)	2	UHPLC-QTOFMS
LPC(16:0p)	2	UHPLC-QTOFMS
LPC(16:1)	2	UHPLC-QTOFMS
LPC(18:0)	1	UHPLC-QTOFMS
LPC(18:1)	1	UHPLC-QTOFMS
LPC(18:2)	1	UHPLC-QTOFMS
LPC(20:3)	2	UHPLC-QTOFMS
LPC(20:4)	1	UHPLC-QTOFMS
LPC(22:6)	1	UHPLC-QTOFMS
PC(16:0/16:0)	1	UHPLC-QTOFMS
PC(16:0/18:1)	2	UHPLC-QTOFMS
PC(16:0e/18:1(9Z))	1	UHPLC-QTOFMS
PC(18:0/18:0)	1	UHPLC-QTOFMS
PC(18:0p/18:1(9Z))	2	UHPLC-QTOFMS
PC(18:0p/22:6)	2	UHPLC-QTOFMS
PC(30:0)	2	UHPLC-QTOFMS
PC(31:0)	2	UHPLC-QTOFMS
PC(31:0)	2	UHPLC-QTOFMS
PC(32:1)	2	UHPLC-QTOFMS
PC(32:2)	2	UHPLC-QTOFMS
PC(33:0)	2	UHPLC-QTOFMS
PC(33:0)	2	UHPLC-QTOFMS
PC(33:1)	2	UHPLC-QTOFMS

PC(34:2)	2	UHPLC-QTOFMS
PC(34:3)	2	UHPLC-QTOFMS
PC(34:3)	2	UHPLC-QTOFMS
PC(35:1)	2	UHPLC-QTOFMS
PC(35:2)	2	UHPLC-QTOFMS
PC(35:3)	2	UHPLC-QTOFMS
PC(35:3)	2	UHPLC-QTOFMS
PC(35:4)	2	UHPLC-QTOFMS
PC(35:4)	2	UHPLC-QTOFMS
PC(35:4)	2	UHPLC-QTOFMS
PC(36:1)	2	UHPLC-QTOFMS
PC(36:2)	2	UHPLC-QTOFMS
PC(36:2)	2	UHPLC-QTOFMS
PC(36:3)	2	UHPLC-QTOFMS
PC(36:3)	2	UHPLC-QTOFMS
PC(36:3)	2	UHPLC-QTOFMS
PC(36:4)	2	UHPLC-QTOFMS
PC(36:4)	2	UHPLC-QTOFMS
PC(36:4)	2	UHPLC-QTOFMS
PC(36:4)	2	UHPLC-QTOFMS
PC(36:5)	2	UHPLC-QTOFMS
PC(36:5)	2	UHPLC-QTOFMS
PC(37:1)	2	UHPLC-QTOFMS
PC(37:2)	2	UHPLC-QTOFMS
PC(37:3)	2	UHPLC-QTOFMS
PC(37:3)	2	UHPLC-QTOFMS
PC(37:5)	2	UHPLC-QTOFMS
PC(37:5)	2	UHPLC-QTOFMS
PC(38:2)	2	UHPLC-QTOFMS
PC(38:2)	2	UHPLC-QTOFMS
PC(38:3)	2	UHPLC-QTOFMS
PC(38:3)	2	UHPLC-QTOFMS
PC(38:4)	2	UHPLC-QTOFMS
PC(38:4)	2	UHPLC-QTOFMS
PC(38:4)	2	UHPLC-QTOFMS
PC(38:5)	2	UHPLC-QTOFMS
PC(38:6)	2	UHPLC-QTOFMS
PC(38:6)	2	UHPLC-QTOFMS
PC(39:6)	2	UHPLC-QTOFMS
PC(39:6)	2	UHPLC-QTOFMS
PC(40:4)	2	UHPLC-QTOFMS

PC(40:4)	2	UHPLC-QTOFMS
PC(40:5)	2	UHPLC-QTOFMS
PC(40:5)	2	UHPLC-QTOFMS
PC(40:5)	2	UHPLC-QTOFMS
PC(40:6)	2	UHPLC-QTOFMS
PC(40:6)	2	UHPLC-QTOFMS
PC(40:6)	2	UHPLC-QTOFMS
PC(40:7)	2	UHPLC-QTOFMS
PC(40:7)	2	UHPLC-QTOFMS
PC(40:8)	2	UHPLC-QTOFMS
PC(40:8)	2	UHPLC-QTOFMS
PC(42:5)	2	UHPLC-QTOFMS
PC(42:6)	2	UHPLC-QTOFMS
PC(O-22:2/22:3)	2	UHPLC-QTOFMS
PC(O-32:0)	2	UHPLC-QTOFMS
PC(O-32:1)	2	UHPLC-QTOFMS
PC(O-34:2)	2	UHPLC-QTOFMS
PC(O-34:3)	2	UHPLC-QTOFMS
PC(O-36:3)	2	UHPLC-QTOFMS
PC(O-36:3)	2	UHPLC-QTOFMS
PC(O-36:3)	2	UHPLC-QTOFMS
PC(O-36:3)	2	UHPLC-QTOFMS
PC(O-36:4)	2	UHPLC-QTOFMS
PC(O-36:4)	2	UHPLC-QTOFMS
PC(O-36:4)	2	UHPLC-QTOFMS
PC(O-36:5)	2	UHPLC-QTOFMS
PC(O-38:4)	2	UHPLC-QTOFMS
PC(O-38:4)	2	UHPLC-QTOFMS
PC(O-38:4)	2	UHPLC-QTOFMS
PC(O-38:5)	2	UHPLC-QTOFMS
PC(O-38:5)	2	UHPLC-QTOFMS
PC(O-38:5)	2	UHPLC-QTOFMS
PC(O-38:6)	2	UHPLC-QTOFMS
PC(O-38:6)	2	UHPLC-QTOFMS
PC(O-38:6)	2	UHPLC-QTOFMS
PC(O-40:4)	2	UHPLC-QTOFMS
PC(O-40:4)	2	UHPLC-QTOFMS
PC(O-40:5)	2	UHPLC-QTOFMS
PC(O-40:5)	2	UHPLC-QTOFMS
PC(O-40:6)	2	UHPLC-QTOFMS
PC(O-40:6)	2	UHPLC-QTOFMS

PC(O-40:6)	2	UHPLC-QTOFMS
PC(P-18:0/22:6)	2	UHPLC-QTOFMS
PE(16:0/18:1)	1	UHPLC-QTOFMS
PE(34:2)	2	UHPLC-QTOFMS
PE(36:4)	2	UHPLC-QTOFMS
PE(38:4)	2	UHPLC-QTOFMS
PE(38:6)	2	UHPLC-QTOFMS
PE(O-16:0/22:6) or PE(P-18:0/20:5)	2	UHPLC-QTOFMS
PE(O-16:0/22:6) or PE(P-18:0/20:5)	2	UHPLC-QTOFMS
PE(O-38:5) or PE(P-38:4)	2	UHPLC-QTOFMS
PE(O-38:5) or PE(P-38:4)	2	UHPLC-QTOFMS
PE(P-18:0/22:6)	2	UHPLC-QTOFMS
PI(18:0/20:4)	2	UHPLC-QTOFMS
SM(d16:1/18:1) or SM(d18:2/16:0)	2	UHPLC-QTOFMS
SM(d18:0/14:0)	1	UHPLC-QTOFMS
SM(d18:0/16:0)	1	UHPLC-QTOFMS
SM(d18:0/16:0)	1	UHPLC-QTOFMS
SM(d18:0/22:0)	2	UHPLC-QTOFMS
SM(d18:1/12:0)	2	UHPLC-QTOFMS
SM(d18:1/24:0)	1	UHPLC-QTOFMS
SM(d18:2/14:0)	2	UHPLC-QTOFMS
SM(d18:2/18:1)	2	UHPLC-QTOFMS
SM(d32:1)	2	UHPLC-QTOFMS
SM(d33:1)	2	UHPLC-QTOFMS
SM(d34:1)	2	UHPLC-QTOFMS
SM(d36:0)	2	UHPLC-QTOFMS
SM(d36:0)	2	UHPLC-QTOFMS
SM(d36:1)	2	UHPLC-QTOFMS
SM(d36:2)	2	UHPLC-QTOFMS
SM(d38:1)	2	UHPLC-QTOFMS
SM(d38:2)	2	UHPLC-QTOFMS
SM(d40:1)	2	UHPLC-QTOFMS
SM(d41:1)	2	UHPLC-QTOFMS
SM(d42:2)	2	UHPLC-QTOFMS
TG(14:0/16:0/18:1)	2	UHPLC-QTOFMS
TG(14:0/18:1/18:1)	2	UHPLC-QTOFMS
TG(14:0/18:2/18:2)	2	UHPLC-QTOFMS
TG(16:0/16:0/16:0)	1	UHPLC-QTOFMS
TG(16:0/18:2/18:2)	2	UHPLC-QTOFMS
TG(16:0/18:2/18:2)	2	UHPLC-QTOFMS
TG(16:0/18:2/18:3)	2	UHPLC-QTOFMS

TG(16:0/18:2/22:6)	2	UHPLC-QTOFMS
TG(16:0/18:2/22:6)	2	UHPLC-QTOFMS
TG(16:0/22:5/18:1) or TG(20:4/18:1/18:1)	2	UHPLC-QTOFMS
TG(16:0/22:5/18:1) or TG(20:4/18:1/18:1)	2	UHPLC-QTOFMS
TG(18:0/18:0/18:0)	1	UHPLC-QTOFMS
TG(18:0/18:1/20:4)	2	UHPLC-QTOFMS
TG(18:0/18:1/20:4)	2	UHPLC-QTOFMS
TG(18:0/18:1/20:4)	2	UHPLC-QTOFMS
TG(18:0/18:1/20:4)	2	UHPLC-QTOFMS
TG(18:1/12:0/18:1) or TG(18:2/16:0/14:0)	2	UHPLC-QTOFMS
TG(18:1/18:1/16:0)	2	UHPLC-QTOFMS
TG(18:1/18:1/18:1)	2	UHPLC-QTOFMS
TG(18:1/18:1/18:1)	2	UHPLC-QTOFMS
TG(18:1/18:1/22:6)	2	UHPLC-QTOFMS
TG(18:1/18:2/18:2)	2	UHPLC-QTOFMS
TG(18:1/18:2/18:2)	2	UHPLC-QTOFMS
TG(18:2/18:1/16:0)	2	UHPLC-QTOFMS
TG(18:2/18:1/16:0)	2	UHPLC-QTOFMS
TG(18:2/18:1/18:1)	2	UHPLC-QTOFMS
TG(18:2/18:1/18:1)	2	UHPLC-QTOFMS
TG(18:2/18:1/18:1)	2	UHPLC-QTOFMS
TG(18:2/18:2/18:2) or TG(18:3/18:2/18:1)	2	UHPLC-QTOFMS
TG(18:2/22:5/16:0)	2	UHPLC-QTOFMS
TG(18:2/22:5/16:0)	2	UHPLC-QTOFMS
TG(18:2/22:5/16:0)	2	UHPLC-QTOFMS
TG(38:1)	2	UHPLC-QTOFMS
TG(45:0)	2	UHPLC-QTOFMS
TG(47:0)	2	UHPLC-QTOFMS
TG(47:0)	2	UHPLC-QTOFMS
TG(47:1)	2	UHPLC-QTOFMS
TG(47:1)	2	UHPLC-QTOFMS
TG(47:2)	2	UHPLC-QTOFMS
TG(48:0)	2	UHPLC-QTOFMS
TG(48:1)	2	UHPLC-QTOFMS
TG(48:1)	2	UHPLC-QTOFMS
TG(48:3)	2	UHPLC-QTOFMS
TG(49:1)	2	UHPLC-QTOFMS
TG(49:2)	2	UHPLC-QTOFMS
TG(49:3)	2	UHPLC-QTOFMS
TG(49:3)	2	UHPLC-QTOFMS
TG(49:3)	2	UHPLC-QTOFMS

TG(50:0)	2	UHPLC-QTOFMS
TG(50:0)	2	UHPLC-QTOFMS
TG(50:0)	2	UHPLC-QTOFMS
TG(50:1)	2	UHPLC-QTOFMS
TG(50:1)	2	UHPLC-QTOFMS
TG(50:2)	2	UHPLC-QTOFMS
TG(50:3)	2	UHPLC-QTOFMS
TG(50:3)	2	UHPLC-QTOFMS
TG(50:3)	2	UHPLC-QTOFMS
TG(50:3)	2	UHPLC-QTOFMS
TG(50:5)	2	UHPLC-QTOFMS
TG(50:5)	2	UHPLC-QTOFMS
TG(51:1)	2	UHPLC-QTOFMS
TG(51:2)	2	UHPLC-QTOFMS
TG(51:2)	2	UHPLC-QTOFMS
TG(51:3)	2	UHPLC-QTOFMS
TG(51:3)	2	UHPLC-QTOFMS
TG(51:4)	2	UHPLC-QTOFMS
TG(51:4)	2	UHPLC-QTOFMS
TG(52:0)	2	UHPLC-QTOFMS
TG(52:2)	2	UHPLC-QTOFMS
TG(52:3)	2	UHPLC-QTOFMS
TG(52:3)	2	UHPLC-QTOFMS
TG(52:4)	2	UHPLC-QTOFMS
TG(52:4)	2	UHPLC-QTOFMS
TG(52:5)	2	UHPLC-QTOFMS
TG(52:6)	2	UHPLC-QTOFMS
TG(53:3)	2	UHPLC-QTOFMS
TG(53:4)	2	UHPLC-QTOFMS
TG(53:4)	2	UHPLC-QTOFMS
TG(53:5)	2	UHPLC-QTOFMS
TG(54:1)	2	UHPLC-QTOFMS
TG(54:2)	2	UHPLC-QTOFMS
TG(54:3)	2	UHPLC-QTOFMS
TG(54:4)	2	UHPLC-QTOFMS
TG(54:4)	2	UHPLC-QTOFMS
TG(54:4)	2	UHPLC-QTOFMS
TG(54:5)	2	UHPLC-QTOFMS
TG(54:5)	2	UHPLC-QTOFMS
TG(54:5)	2	UHPLC-QTOFMS
TG(54:5)	2	UHPLC-QTOFMS

TG(54:6)	2	UHPLC-QTOFMS
TG(54:6)	2	UHPLC-QTOFMS
TG(54:7)	2	UHPLC-QTOFMS
TG(54:7)	2	UHPLC-QTOFMS
TG(54:7)	2	UHPLC-QTOFMS
TG(54:7)	2	UHPLC-QTOFMS
TG(55:5)	2	UHPLC-QTOFMS
TG(56:2)	2	UHPLC-QTOFMS
TG(56:3)	2	UHPLC-QTOFMS
TG(56:4)	2	UHPLC-QTOFMS
TG(56:4)	2	UHPLC-QTOFMS
TG(56:4)	2	UHPLC-QTOFMS
TG(56:4)	2	UHPLC-QTOFMS
TG(56:5)	2	UHPLC-QTOFMS
TG(56:5)	2	UHPLC-QTOFMS
TG(56:6)	2	UHPLC-QTOFMS
TG(56:6)	2	UHPLC-QTOFMS
TG(56:6)	2	UHPLC-QTOFMS
TG(56:7)	2	UHPLC-QTOFMS
TG(56:9)	2	UHPLC-QTOFMS
TG(58:6)	2	UHPLC-QTOFMS
TG(58:6)	2	UHPLC-QTOFMS
TG(58:9)	2	UHPLC-QTOFMS
TG(60:7)	2	UHPLC-QTOFMS
TG(60:7)	2	UHPLC-QTOFMS
decanoylcarnitine	1	UHPLC-QTOFMS
hexadecenoylcarnitine	1	UHPLC-QTOFMS
Hexanoylcarnitine	1	UHPLC-QTOFMS
L-Carnitine	1	UHPLC-QTOFMS
octadecenoylcarnitine	1	UHPLC-QTOFMS
Palmitoylcarnitine	1	UHPLC-QTOFMS
stearoylcarnitine	1	UHPLC-QTOFMS
